# Editorial: Checkpoint immunotherapy: reshaping the landscape of gastrointestinal cancer treatment, volume II

**DOI:** 10.3389/fimmu.2026.1943344

**Published:** 2026-08-05

**Authors:** Stavros P. Papadakos, Charalampos Theocharopoulos, Elias Kouroumalis, Stamatios E. Theocharis, Georgios Germanidis

**Affiliations:** 1 Department of Gastroenterology, Laiko General Hospital, National and Kapodistrian University of Athens, Athens, Greece; 2 Department of Surgery, University of Colorado Anschutz Medical Campus, Aurora, CO, United States; 3 Laboratory of Gastroenterology and Hepatology, School of Medicine, University of Crete, Heraklion, Greece; 4 First Department of Pathology, School of Medicine, National and Kapodistrian University of Athens, Athens, Greece; 5 AHEPA University Hospital, Aristotle University of Thessaloniki, Thessaloniki, Greece

**Keywords:** biomarker, gastrointestinal cancer, immune check inhibitor (ICI), immunotherapy, tumor micro environment (TME)

Immune checkpoint inhibitors (ICIs) have achieved notable successes in selected gastrointestinal cancers and have reshaped modern treatment algorithms. The clearest benefits have been observed in mismatch repair-deficient and microsatellite instability-high (dMMR/MSI-H) colorectal cancer, esophageal carcinoma, gastric/gastroesophageal adenocarcinoma and hepatocellular carcinoma (HCC) (1). In contrast, most microsatellite-stable colorectal cancers, pancreatic cancers, and many biliary tract cancers remain poorly responsive to ICI monotherapy, underscoring the need for biomarker-guided patient selection and combination with multimodal therapeutics to achieve a meaningful benefit (2). The manuscripts published in this Research Topic reflect the evolution from empirical use of ICIs toward biologically informed combination strategies, improved patient selection, integration of ICIs in multimodality regimens and a deeper understanding of mechanisms of resistance.

In HCC, authors addressed important questions about ICI-based therapy: the efficacy and safety of first-line combinations, the role of locoregional intensification, the cost-effectiveness of newer regimens, and the prognostic value of systemic inflammatory markers after surgery. Lin et al. pooled eight randomized phase III trials including 4,379 patients with unresectable/advanced HCC. Compared with sorafenib or lenvatinib monotherapy, first-line ICI–targeted therapy combinations increased the odds of objective response (OR 3.93) and reduced the hazards of death and progression/death (overall survival HR: 0.71; progression-free survival HR: 0.62) (3). However, this survival benefit was accompanied by an approximately doubled risk of serious treatment-related adverse events. Ma et al. reported the outcomes of a four-component regimen combining DEB-TACE, HAIC, donafenib, and camrelizumab in patients with unresectable hepatocellular carcinoma. In this multicenter retrospective study of 122 patients, the DEB-TACE-HAIC plus donafenib and camrelizumab group had higher objective response and disease control rates than the DEB-TACE plus donafenib and camrelizumab group, both before and after inverse probability of treatment weighting (4). The HAIC-containing regimen was also associated with longer median progression-free survival and overall survival, while portal vein tumor thrombosis and extrahepatic metastasis were independent predictors of poorer outcomes. Li et al. evaluated the cost-effectiveness of toripalimab plus bevacizumab versus sorafenib as first-line treatment for advanced HCC in China, using HEPATORCH trial data in a three-state partitioned survival model (5). Toripalimab plus bevacizumab yielded higher life-years and quality-adjusted life-years at greater cost, with an incremental cost-effectiveness ratio (ICER) of $24,602.67/QALY, which was below the prespecified willingness-to-pay threshold; sensitivity analyses identified bevacizumab cost and receipt of subsequent lines of treatment in the sorafenib arm as key influential parameters on the ICER. Arvanitakis et al. evaluated serial inflammatory biomarkers in a multicenter retrospective cohort of 74 HCC patients undergoing curative-intent liver resection or orthotopic liver transplantation (6). Postoperative neutrophil-to-lymphocyte ratio (NLR) independently predicted overall survival after adjustment for surgical procedure and BCLC stage. Preoperative platelet to lymphocyte ratio (PLR) predicted major postoperative complications, and 12-month PLR provided additional prognostic information. These findings support NLR and PLR as accessible adjuncts for postoperative risk stratification, pending prospective validation.

For gastric and gastroesophageal junction cancer, the included articles examine variable ICI responsiveness across disease settings and the need for better biomarkers, safer perioperative strategies, and rational combinations. Liu et al. reviewed the role of ICI across first-line, later-line, perioperative, HER2-positive, and combination settings. They emphasized that efficacy remains constrained by low monotherapy activity in unselected patients, primary and acquired resistance, immune-related adverse events, insufficiently reliable predictive biomarkers, and an immunosuppressive tumor microenvironment (7). Priorities for precision immunotherapy research include biomarker discovery, predictive modeling, optimized combination regimens, microbiota modulation, resistance targeting, and toxicity management. Cai et al. reviewed perioperative immunotherapy for resectable gastric/GEJ adenocarcinoma across unselected, HER2-positive, and dMMR/MSI-H populations. MATTERHORN showed that durvalumab plus FLOT improved pathologic complete response (pCR) and event-free survival (EFS), whereas DRAGON IV/CAP-05 increased pathologic complete response with camrelizumab, rivoceranib, and SOX (8). Conversely, KEYNOTE-585 missed its EFS endpoint despite higher pCR, while negative ATTRACTION-5 and VESTIGE results caution against routine postoperative escalation. Zhan et al. conducted a prospective single-center phase II trial of perioperative serplulimab plus SOX in 33 patients with resectable locally advanced gastric/GEJ adenocarcinoma (9). After up to three neoadjuvant cycles, all patients underwent D2 gastrectomy with R0 resection; pCR and MPR were 21.21% and 36.36%, with a 12-month EFS rate of 82.20%. MPR correlated with higher preoperative IL-1β, lower CD4+/Treg ratio, and higher Treg/CD8 ratio. Exploratory parenteral-nutrition analysis showed transient preoperative immune/inflammatory effects without improved pathological response. Li et al. described a single-arm phase Ib/II protocol evaluating hyperbaric oxygen (HBOT) in combination with XELOX and sintilimab in HER2-negative advanced gastric/GEJ adenocarcinoma (10). The rationale is that tumor hypoxia limits checkpoint-inhibitor efficacy by promoting extracellular-matrix remodeling, impairing perfusion and drug delivery, reducing cytotoxic T-cell infiltration/function, and recruiting Tregs, MDSCs, and M2 macrophages. Hyperbaric oxygen is proposed to reverse these barriers and sensitize tumors to PD-1 blockade. Phase Ib will optimize the HBOT regimen, while phase II will assess ORR. Yu and Zhu described a durable response in recurrent EBV-associated metastatic gastric adenocarcinoma treated with CAPOX plus nivolumab and TST001 (11). The tumor was HER2-negative, MMR-proficient, PD-L1 combined positive score 3, and CLDN18.2-low. Regression emerged after two cycles and continued for 19 months after treatment cessation for an aortic dissection, culminating in a near-complete radiographic response. This prolonged treatment-free disease control is clinically notable given the low CLDN18.2 expression and limited conventional predictors of checkpoint sensitivity.

Articles on esophageal squamous cell carcinoma (ESCC) examined how neoadjuvant immunotherapy influences pathological response, treatment-related toxicity, and patterns of residual nodal disease at resection. Zheng et al. randomized 70 patients with resectable locally advanced ESCC to sequential or concurrent neoadjuvant toripalimab with paclitaxel–cisplatin (12). Among 54 patients undergoing surgery, all achieved R0 resection, while pCR, disease free survival, and OS did not differ significantly. Importantly, concurrent immunochemotherapy caused more nausea and diarrhea and accounted for five of six treatment-related deaths, raising an important safety concern. Han et al. retrospectively analyzed 208 patients with locally advanced ESCC undergoing esophagectomy after neoadjuvant chemoradiotherapy with or without pembrolizumab (13). Pembrolizumab was associated with higher major pathological response (79.5% vs 65.4%) but not pathological complete response, and lower metastasis rates at three nodal stations. These findings suggest enhanced tumor and nodal regression, however the single-center, nonrandomized design limits generalizability and necessitates further evaluation.

The central challenge in colorectal cancer immunotherapy remains the microsatellite-stable subset, which derives little benefit from checkpoint blockade. The contributions in this Research Topic examine the mechanisms that shape immune resistance and response in colorectal cancer, including cancer stemness, alternative checkpoint expression, PIN1-driven microenvironmental remodeling, cGAS–STING signaling, and the relationship between antitumor immunity and treatment-related intestinal inflammation. Hussein et al. explored the immunologic phenotype of colorectal cancer stem-like cells by integrating spheroid-based experiments with transcriptomic analyses of the TCGA-COAD cohort (14). Stem-cell enrichment in HCT-116 and SW620 spheroids was accompanied by increased expression of several inhibitory checkpoints, most consistently PD-L1 and B7-H3. Notably, higher B7-H3 mRNA expression in an external colorectal cancer survival dataset was associated with poorer overall survival. The TCGA analyses revealed a more complex immunologic phenotype: high-stemness tumors exhibited lower overall immune and stromal scores but higher TMB and a greater prevalence of MSI-high disease, suggesting that an immune-excluded microenvironment may coexist with molecular features potentially associated with checkpoint responsiveness. These findings identify cancer stem-like cells as potential contributors to checkpoint-mediated immune evasion and highlight B7-H3 as a candidate therapeutic target, in line with its emerging development as an immunotherapy target in solid tumors (15). Wang et al. identified PIN1 as a potential mediator of immune exclusion and checkpoint resistance in MSS colorectal cancer by integrating transcriptomic and single-cell analyses with human tissue, cell-line, and murine experiments (16). PIN1 was preferentially expressed in MSS tumors and associated with reduced CD4+ and CD8+ T-cell infiltration and enrichment of immunosuppressive stromal and regulatory-cell populations, although its expression was not significantly associated with overall survival. Mechanistically, PIN1 interacted with p65 and activated NF-κB-dependent CCL3 expression, promoting CCR5-mediated Treg recruitment and CAF activation. In subcutaneous and hepatic-metastasis models, the selective PIN1 inhibitor sulfopin enhanced the antitumor activity of anti-PD-1 therapy, reduced Treg infiltration and CAF activation, and increased effector T-cell accumulation (16). These findings position PIN1 as a candidate microenvironment-remodeling target for overcoming immunotherapy resistance in MSS colorectal cancer. Chen et al. reviewed cGAS–STING signaling as a context-dependent regulator of intestinal homeostasis, inflammatory bowel disease, and colorectal cancer. Balanced activation preserves barrier integrity, mucosal defense, and antitumor immunity, whereas sustained activation drives chronic inflammation and tissue injury; conversely, impaired signaling may facilitate immune escape and treatment resistance (17). The review frames cGAS–STING modulation as a promising therapeutic strategy while emphasizing unresolved challenges in tissue-specific delivery, pathway selectivity, systemic toxicity, and biomarker-guided patient selection. Xu et al. retrospectively examined anti-PD-1 toxicity in 138 patients with gastrointestinal cancers, including 31 with preexisting ulcerative colitis (UC) (18). Immune-related colitis developed in 71% of the UC cohort, was frequently severe, and often required corticosteroids and treatment discontinuation. Mild colitis was independently associated with improved survival. Elevated IL-6 and IL-17A implicated Th17-associated inflammation. However, the small retrospective cohort, difficulty distinguishing immune-related colitis from UC relapse, and inclusion of only quiescent-to-mildly active UC limit causal interpretation; mild colitis should not yet be regarded as a validated efficacy biomarker. Luo et al. reported a case of a 62-year-old woman with pMMR/MSS, KRAS p.G12D sigmoid colon adenocarcinoma (cT4aN2bM1b) with liver, lung, and nodal metastases who was managed with serplulimab, bevacizumab, and XELOX with excellent outcomes (19). Despite a molecular profile that is generally associated with resistance to ICI, the patient achieved complete radiographic and endoscopic response and remained progression-free at 12 months of follow-up. Notably, the tumor had low TMB and negative PD-L1 expression but substantial baseline CD4+ and CD8+ T-cell infiltration, suggesting that patients with baseline immune-infiltrated TME may be responsive to ICI-based combinations. This case is consistent with findings from the ongoing phase II/III NCT04547166 trial (20) and illustrates the potential efficacy of serplulimab, bevacizumab, and XELOX in a real-world setting.

Benefit from checkpoint blockade in biliary and pancreatic adenocarcinoma remains limited to a minority, and the biomarkers that define it, PD-L1 expression, microsatellite instability, tumor mutational burden, are not universally assessed in practice. The following cases illustrate two different scenarios: a marked response in a molecularly uncharacterized patient and a comparable response in a patient with a favorable molecular profile. Wang et al. described a case of successful conversion of initially unresectable stage IVB (cT3N1M1) gallbladder adenocarcinoma with hepatic metastasis and hepatoduodenal ligament nodal involvement using camrelizumab combined with gemcitabine and cisplatin (21). Following a marked biochemical and radiographic response, the patient successfully underwent open cholecystectomy with segment IVb/V liver resection, lymphadenectomy, and hepaticojejunostomy. Despite receiving no adjuvant systemic therapy, the patient remained recurrence-free on serial imaging 14 months after surgery. However, the absence of PD-L1, MSI and TMB testing precludes identification of the biological basis of this exceptional response and the specific contribution of camrelizumab. Tang et al. described a sustained partial response in a patient with metastatic pancreatic ductal adenocarcinoma, characterized by high TMB, microsatellite instability and KRAS wild-type status (22). Treatment with cadonilimab, nimotuzumab, nab-paclitaxel, and gemcitabine produced marked regression of both the pancreatic primary and multifocal hepatic disease, with progression-free survival exceeding seven months. This molecular profile provides a plausible rationale for this approach, as high TMB may confer sensitivity to dual ICI blockade, whereas KRAS wild-type status provides a biological rationale for EGFR-directed therapy (22). Treatment was generally well tolerated, although grade 2 hypothyroidism and hematologic toxicity were reported.

Two overarching themes emerge from this collection. First, checkpoint inhibition in gastrointestinal cancer is increasingly combination-dependent, integrated with targeted therapy, anti-angiogenic therapy, chemotherapy, locoregional treatment, chemoradiotherapy, hypoxia modulation, and microenvironment-directed approach (23). Second, biomarker assessment must move beyond single markers toward integrated profiles incorporating MSI/MMR status, TMB, KRAS status, angiogenic biology, cancer stemness, stromal composition, innate immune signaling, systemic inflammatory indices, and immune-related toxicity risk (24). Collectively, these studies document a transition from empirical checkpoint inhibitor use toward selective immune modulation, where benefit depends on tumor biology, immune context, treatment timing, patient risk, and healthcare setting.

Summarizing, the contributions in this Research topic highlight two important frontiers: first, that checkpoint inhibition research in gastrointestinal cancers is increasingly combination-dependent; and second, that biomarker assessment is moving toward integrated profiles incorporating MSI/MMR status, TMB, KRAS status, angiogenic biology, cancer stemness, stromal composition, innate immune signaling, systemic inflammatory indices, and immune-related toxicity risk.

The editors of this Research Topic would like to thank all authors for their valuable contributions and reviewers for their thoughtful evaluations and support throughout the review process. We hope that this Research Topic provides meaningful insights into immune checkpoint inhibition in gastrointestinal cancers and contributes to ongoing research and clinical advancement in the field.
